# Suicide Among Older Adults Living in or Transitioning to Residential Long-term Care, 2003 to 2015

**DOI:** 10.1001/jamanetworkopen.2019.5627

**Published:** 2019-06-14

**Authors:** Briana Mezuk, Tomohiro M. Ko, Viktoryia A. Kalesnikava, David Jurgens

**Affiliations:** 1 Institute for Social Research, University of Michigan, Ann Arbor; 2 Department of Epidemiology, University of Michigan School of Public Health, Ann Arbor; 3 School of Information, University of Michigan, Ann Arbor

## Abstract

**Importance:**

Almost 25% of Medicare beneficiaries live in residential long-term care (LTC) (eg, independent or assisted living facility or nursing home). There are few reliable statistics on completed suicide in LTC, in part because of data limitations.

**Objectives:**

To estimate the number of suicides associated with residential LTC (ie, among persons in a facility, transitioning into or out of a facility, or otherwise associated with LTC) among adults 55 and older and, secondarily, to identify whether machine learning tools could improve the quality of suicide surveillance data.

**Design, Setting, and Participants:**

Cross-sectional epidemiologic study (conducted in 2018) of restricted-access data from the National Violent Death Reporting System (NVDRS) (2003-2015) using restricted-access case narratives from suicides and undetermined deaths among adults 55 years and older in 27 states. Participants were all suicides and undetermined deaths (N = 47 759) among persons 55 years and older.

**Exposure:**

Long-term care cited in the coroner/medical examiner case narrative, whether as a reason for self-harm or the injury location, identified using machine learning natural language processing (NLP) algorithms plus manual review of texts.

**Main Outcomes and Measures:**

Number and characteristics (eg, demographics, health history, and means of injury) of suicides associated with LTC. The κ statistic was used to estimate the reliability of the existing NVDRS injury location codes relative to cases identified by the algorithm.

**Results:**

Among 47 759 persons 55 years and older (median age, 64 years; 77.6% male; 90.0% non-Hispanic white), this study identified 1037 suicide deaths associated with LTC, including 428 among older adults living in LTC, 449 among older adults transitioning to LTC, and 160 otherwise associated with LTC. In contrast, there were only 263 cases coded with the existing NVDRS location code “supervised residential facility,” which had poor agreement with cases that the algorithm identified as occurring in LTC (κ statistic, 0.30; 95% CI, 0.26-0.35).

**Conclusions and Relevance:**

Over a 13-year period, approximately 2.2% of suicides among adults 55 years and older were associated with LTC in some manner. Clinicians, administrators, and policy makers should consider ways to promote the mental health and well-being of older adults experiencing functioning limitations and their families. Natural language processing may be a useful way to improve abstraction of variables in the NVDRS.

## Key Points

**Question:**

How common is suicide in residential long-term care (LTC) settings, and is transitioning into or out of LTC associated with suicide among older adults?

**Findings:**

In this cross-sectional epidemiologic study of 47 759 deaths in the National Violent Death Reporting System (NVDRS) (2003-2015), a natural language processing algorithm identified 1037 suicides among adults 55 years and older associated with LTC. The algorithm identified far more deaths associated with LTC in comparison with the existing NVDRS injury location codes.

**Meaning:**

Suicide occurs among older adults living in or transitioning to LTC settings, and the mental health and well-being of older adults should be addressed in these settings.

## Teaser

This cross-sectional epidemiologic study of deaths in the US National Violent Death Reporting System (2003-2015) estimates the number of suicides associated with residential long-term care among adults 55 and older and identifies whether machine learning tools could improve the quality of suicide surveillance data.

## Introduction

Forty million US adults are 65 years and older, and 5.5 million are 85 years and older; by 2050, 1 in 5 will be 65 or older.^1^ Suicide is the 10th leading cause of death in the United States, and the rate of suicide has risen substantially in the past decade, particularly for older adults.^2^ In parallel, there is growing recognition of a lack of progress on identifying clinically meaningful and modifiable predictors of suicide. A recent editorial noted that “…lack of progress in suicide prevention is in large part owing to our limited understanding of this problem…[W]e lack a firm understanding of the fundamental properties of [suicidal thoughts and behaviors], and when, why, and among whom they unfold.”^3^^(p11)^

That statement suggests an additional question: where does suicidal behavior occur? Understanding the location of suicidal behavior can help identify potential “points of engagement” for reducing suicide risk, including before acute crisis.^4^ One location relevant to suicide prevention among older adults is residential long-term care (LTC) (eg, nursing homes, assisted living facilities, and continuing care retirement communities).^5^ While 90% of US adults want to remain living in their own home as they age,^6^ this desire to live independently often does not match the reality of “aging in place.” Only 40% who are 70 years and older report that it is “very easy” to live independently, and 20% say that they cannot do so without occasional assistance.^6,7^

The Centers for Disease Control and Prevention estimates that there are approximately 16 000 nursing homes and 31 000 assisted living facilities in the United States,^8,9^ and almost 25% of Medicare beneficiaries in 2010 were living in these settings.^10^ As such, residential LTC may be a potential location for identifying individuals at high risk of self-harm and for implementing interventions to reduce suicide risk. The Substance Abuse and Mental Health Services Administration recognized the potential for residential LTC to serve as a point of engagement in their 2011 toolkit for practitioners on how to promote emotional health and prevent suicide in these settings.^11^ However, even this toolkit notes that “There are few reliable statistics on suicide in senior living communities.”^11^^(pv)^

Beyond suicides that occur in LTC facilities, the process of transitioning to these settings may also be related to suicide risk. Housing transitions, particularly those that occur in the context of functional impairment, often involve a complex interplay of psychosocial factors, including anxiety, loss of autonomy, and isolation.^12,13,14^ While this is an understudied issue, 2 prior studies^15,16^ have shown that transitioning to LTC can be a precipitating factor for suicide among older adults.

Understanding suicide risk requires accurate data. The National Violent Death Reporting System (NVDRS) is a surveillance system designed to provide information on contextual factors related to suicide and other violent deaths. The NVDRS staff individually abstract textual data from medical examiner and law enforcement reports to generate quantitative variables for each death.^17,18^ Some of this abstracted text is retained in the form of restricted-access case narratives. These narratives have the potential to identify novel risk factors for suicide on a population scale, including the creation of new quantitative variables. Moreover, the current approach to creating quantitative variables (ie, individually abstracted by staff) could be augmented by machine learning tools. Natural language processing (NLP) algorithms provide a means of efficiently categorizing large amounts of complex textual data,^19^ but they have not yet been used in these data to our knowledge.

In this study, we used NLP algorithms to identify suicides associated with residential LTC by analyzing textual narratives of approximately 50 000 decedents 55 years and older from the 27 states that participated in the NVDRS from 2003 to 2015. All analyses in this cross-sectional epidemiologic study were conducted in 2018. We had the following 2 substantive objectives: (1) to estimate the number of suicides associated with residential LTC (ie, people living in LTC, people transitioning into or out of LTC, and otherwise associated with LTC) and (2) to characterize deaths associated with LTC compared with other suicide decedents. An overarching methodologic goal was to identify ways to improve the quality of suicide surveillance data systems like the NVDRS.

## Methods

### NVDRS Restricted-Access Narrative Texts as Data Source

The restricted-access NVDRS data used include all the quantitative variables from the publicly available NVDRS^20^ plus textual narratives (mean [SD] length, 513.8 [344.2] characters) abstracted from the coroner/medical examiner (CME) reports. While the NVDRS also abstracts narratives from law enforcement reports, these were not used due to the large amount of blank narratives from this source. Our study followed the Strengthening the Reporting of Observational Studies in Epidemiology (STROBE) reporting guideline. The request for restricted-access narratives was approved by the Centers for Disease Control and Prevention, and this study was determined to be exempt from human participant regulations because all participants were deceased at the time of data collection.

The analytic sample consisted of 47 759 deaths (median age, 64 years; 77.6% male; 90.0% non-Hispanic white), including 42 576 suicides, 279 deaths due to unintentional firearm injury, and 4904 undetermined deaths among adults 55 years and older recorded in the NVDRS from 2003 to 2015. Accidental and undetermined deaths were included because suicide is often misclassified.^21,22^ Details about NVDRS data collection and quality control/assurance procedures are available elsewhere.^17,20^ The eAppendix and eTable 1 in the Supplement provide details on individual states, including years represented in the NVDRS and CME system. Deaths missing CME narratives (n = 2578) were excluded. eTable 2 in the Supplement compares the analytic sample with cases excluded due to missing narratives.

### Quantitative Characteristics

The NVDRS quantitative variables were recorded. These included the following: demographic characteristics (ie, age, sex, race/ethnicity [recoded as non-Hispanic white vs other for this analysis], and marital status [married/in a relationship, single/never married, widowed, or divorced/separated]), injury location (see below), health status (ie, ongoing physical health problem) (yes or no), history of suicidal ideation (yes or no), current depressed mood (yes or no), whether the decedent had a recent crisis (eg, conflict with family, finances, etc) (yes or no), death location (eg, hospital, private home, etc), and means of injury (eg, firearm, poisoning, fall, etc).

### Analysis

#### Identifying Suicides Related to Residential LTC

The NVDRS has 2 variables that indicate location, including (1) injury location (ie, where the decedent was when he or she self-harmed) and (2) death location (ie, where the decedent was when he or she died, which may be different from the injury location) (eg, if he or she was found and transported to a hospital). There is no injury location variable specific for residential LTC; the best approximation is the code “supervised residential facility” (SRF). A total of 263 suicide injuries were coded as SRF in our analytic sample. A death location code exists for “LTC/nursing home,” and 569 suicide deaths had this code in our analytic sample.

However, there are 4 limitations with these existing variables. First, the SRF code is not specific to LTC because it broadly encompasses “group homes.” Second, prior work using the Virginia site of the NVDRS showed that the SRF injury location code has poor (<50%) sensitivity in identifying suicides in residential LTC relative to geocode-confirmed locations.^15^ Third, the LTC/nursing home death location code does not mean that the self-harm occurred in that setting; if someone attempted suicide at home, survived, was transferred to LTC, and then died there, even several years later, this would be included in this code. Fourth, neither location code will identify persons in the process of transitioning to LTC when they died or persons whose death was otherwise related to LTC (eg, among caregivers of a person in LTC, among persons concerned about the financial strain of LTC, or among persons anxious about the possibility of needing LTC, even if they were not actively in the process of making this transition) because these deaths will likely not have occurred in a facility. Therefore, for this analysis, these location codes are used only as a means of comparison with the NLP algorithm.

#### Analysis of NVDRS Text Narratives Using Machine Learning Algorithms

We developed a supervised machine learning algorithm to identify suicides related to LTC using the CME narratives; we treat this issue as a classification problem in trying to distinguish narratives associated with LTC from those that are not.^23^ We used NLP techniques to convert the text narratives into computationally analyzable representations; these techniques are used to solve large-scale text classification problems, such as spam recognition, and are beginning to be used in health care.^24,25^ The process of developing, training, iterating, and cross-validating the algorithm is summarized below and detailed in eFigure 1 in the Supplement.

##### (1) Create the Initial Training (Labeled) Data Set Using Keywords

We began the process of generating an initial training data set using keywords selected based on prior knowledge (eg, *long-term*, *convalescent*, and *residential*) to search the narratives; these keywords identified 7806 narratives potentially related to LTC. Two raters (T.M.K. and V.A.K.) annotated a random sample of these narratives to construct the initial labeled data set, which consisted of 103 deaths associated with LTC (“true positives”) and 264 deaths not associated with LTC (“true negatives”) to train the classifier.

##### (2) Train and Update the NLP Algorithm

This initial labeled data set was used to develop a supervised machine learning classifier to identify additional true-positive cases associated with LTC and true-negative cases not associated with LTC. The narratives were converted into a matrix of rows corresponding to narratives and columns corresponding to the features of each narrative using standard NLP procedures.^25^ Next, a random forest NLP classifier was trained to predict the status (associated with LTC or not) of the remaining unlabeled data from this vector of features.^26^ This classifier consisted of 1500 decision trees that were each trained from a random subset of the data to prevent model overfitting.^27^ Standard metrics of cross-validation were used to quantify the performance of this algorithm,^26^ and performance was consistently high. The classifier assigned each unlabeled narrative a probability that it was associated with LTC. At this step, the algorithm identified 943 additional narratives potentially associated with LTC that had a probability exceeding 0.50.

Next, we manually annotated a random sample of 41 narratives potentially associated with LTC to iteratively update the training data set. We selected this sample from across the total case probability distribution (from 0.10 to 0.90). All 4 of us independently reviewed these narratives and classified them as being associated with LTC or not, with high interrater agreement (κ = 0.69). Discordant cases were adjudicated by consensus, with a final determination of 14 cases associated with LTC and 27 noncases. These newly labeled cases and noncases were then added to the training set. By providing this type of iterative feedback, especially for cases with probabilities near 0.50, the algorithm learned the features (ie, types of phrases and context and structure of phrases) of otherwise ambiguous narratives associated with LTC.

##### (3) Finalize the NLP Algorithm and Characterize Cases

Step 2 was repeated with the updated labeled training set (eFigure 2 in the Supplement). Using a threshold probability of greater than 0.50, the algorithm identified 1200 suicide deaths potentially associated with LTC. The cross-validation performance of the classifier remained high, indicating that the algorithm gained ability to identify true-positive status of previously ambiguous instances, without a decrease in performance.

With the NLP model finalized, all cases with probability of 0.50 or less were assigned a status of “not associated with LTC.” Next, 2 raters (T.M.K. and V.A.K.) each manually annotated 50% (n = 600) of the cases with probability exceeding 0.50 identified by the final NLP model and classified them as one of the following 4 groups. First, the decedent was residing in residential LTC at the time of his or her injury (n = 331). Second, the decedent was in the process of transitioning to (or out of) LTC (n = 432). Third, the decedent was otherwise associated with LTC (ie, LTC was cited as a salient factor in his or her death, but the decedent was not in the first or second groups (n = 157). This group included decedents who were caring for a family member who was living in LTC, had experienced a recent hospitalization that gave rise to fears that he or she may need LTC but there was no indication that this transition was actually happening at the time of death, or had expressed concerns about the financial burden of LTC for themselves or a loved one (n = 157). Fourth, the decedent was not associated with LTC (n = 280) (ie, false-positives). In all instances, the decedent narratives classified as the first, second, or third groups explicitly referenced LTC as a salient circumstance or precipitating event of the death.

**Figure 1.**
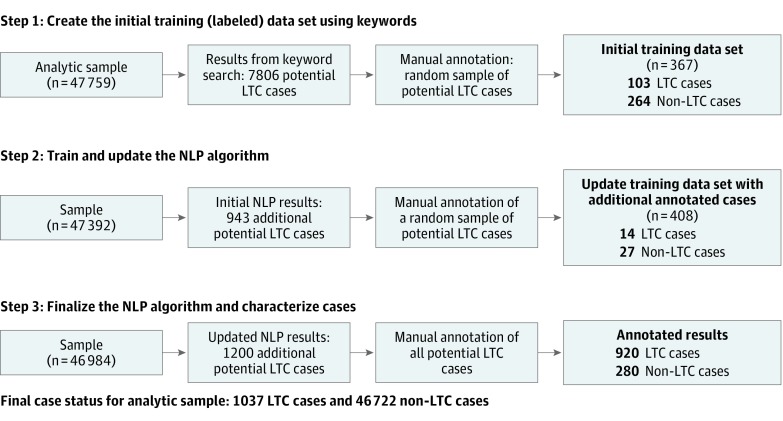
Flowchart of the Development and Training of the Natural Language Processing (NLP) Algorithm to Identify Cases Associated With Long-term Care (LTC), 2003 to 2015 Stepwise development and training used coroner/medical examiner narratives of decedents 55 years and older (N = 47 759) in the National Violent Death Reporting System.

Figure 1 and eTable 3 in the Supplement summarize how these 4 groups of cases were identified at each step of the NLP process. eTable 4 in the Supplement lists heuristic examples of narratives from each of these case groups. The annotated cases were then combined with the rest of the analytic sample to characterize all deaths.

### Statistical Analysis

We characterized all decedents in our analytic sample using existing NVDRS variables (eg, demographic characteristics, health and psychosocial circumstances, and injury characteristics). The performance of the NLP algorithm compared with the SRF injury location and the LTC/nursing home death location was evaluated using the κ statistic. We used Poisson regression to assess whether the number of suicides associated with LTC varied from 2005 to 2015; we limited this analysis to the 13 states that began reporting to the NVDRS in 2003 or 2004 to reduce the possibility of temporal artifacts of the relative inexperience of narrative abstractors from the more recently included states.

The NLP algorithm was developed using Python (version 3.7.0; Python Software Foundation) and Scikit-Learn (version 20.1; Python Software Foundation).^25^ Statistical analyses were conducted using R (versions 3.4.3 and 3.4.4; R Foundation for Statistical Computing). All *P* values refer to 2-tailed α = .05. Analyses of variance were used to determine group differences in means for continuous measures, whereas χ^2^ tests were used to determine associations for categorical measures.

## Results

In total, summing across the training and testing data, we identified 1037 suicides or undetermined deaths associated with LTC among adults 55 years and older. Decedents whose death was associated with LTC, representing 2.2% of decedents, had a median age of 79 years, 73.8% were male, and 94.3% were non-Hispanic white. Table 1 further summarizes characteristics of decedents by whether their death occurred when they were (1) living in an LTC facility (n = 428), (2) transitioning into or out of LTC (n = 449), (3) otherwise associated with LTC (n = 160), or (4) not associated with LTC (n = 46 722). Deaths that occurred in LTC were more likely to be among women, which is expected given that LTC residents, in general, are disproportionately female. Decedents in transition to or out of LTC were more likely to have expressed suicidal ideation and were more likely to have a physical health problem cited as a contributing circumstance relative to those living in LTC. Decedents whose death was otherwise associated with LTC were more likely to be married/in a relationship, have depressed mood, and have had a recent crisis cited as a contributing factor relative to the other LTC groups.

**Table 1.** Decedent Characteristics Stratified by Association With LTC as Identified by the Natural Language Processing Algorithm (National Violent Death Reporting System, 2003 to 2015)

Variable	All Deaths Not Associated With LTC (n = 46 722)	All Deaths Associated With LTC (n = 1037)	Living in LTC (n = 428)	Transitioning Into or Out of LTC (n = 449)	Otherwise Associated With LTC (n = 160)	χ^2^ Statistic	*df*^a^	*P* Value^a^
**Demographic Characteristics**								
Age, median (IQR), y	64 (15)	79 (17)	79 (19)	80 (13)	75 (20)	67	46	.02
Male, No. (%)	36 230 (77.6)	765 (73.8)	277 (64.7)	360 (80.2)	128 (80.0)	31	2	<.001
Non-Hispanic white, No. (%)	41 983 (89.8)	978 (94.3)	396 (92.5)	428 (95.3)	154 (96.2)	5	2	.10
Education, No. (%)								
<High school	5199 (11.1)	126 (12.2)	41 (9.6)	64 (14.3)	21 (13.1)	13	6	.04^b^
High school or GED	11 624 (24.9)	255 (24.6)	93 (21.7)	116 (25.8)	46 (28.7)			
>High school	12 122 (25.9)	256 (24.7)	114 (26.6)	112 (24.9)	30 (18.8)			
Unknown	16 065 (34.4)	360 (34.7)	165 (38.6)	139 (31.0)	56 (35.0)			
Missing	1712 (3.7)	40 (3.9)	15 (3.5)	18 (4.0)	7 (4.4)			
Marital status, No. (%)								
Married/in a relationship	20 687 (44.3)	333 (32.1)	100 (23.4)	117 (26.1)	116 (72.5)	162	8	<.001^b^
Single/never married	5067 (10.8)	98 (9.5)	58 (13.6)	28 (6.2)	12 (7.5)			
Widowed	7117 (15.2)	386 (37.2)	168 (39.3)	204 (45.4)	14 (8.8)			
Divorced/separated	13 047 (27.9)	214 (20.6)	99 (23.1)	98 (21.8)	17 (10.6)			
Unknown	759 (1.6)	S	S	S	S			
Missing	45 (0.1)	S	S	S	S			
**Health and Psychosocial Circumstances, No. (%)**								
Depressed mood	16 789 (35.9)	468 (45.1)	168 (39.3)	202 (45.0)	98 (61.3)	23	2	<.001
Physical health problem	17 632 (37.7)	632 (60.9)	217 (50.7)	351 (78.2)	64 (40.0)	104	2	<.001
History of suicide ideation	5619 (12.0)	130 (12.5)	40 (9.3)	76 (16.9)	14 (8.8)	14	2	<.001
Recent crisis	5272 (11.3)	205 (19.8)	51 (11.9)	121 (26.9)	33 (20.6)	31	2	<.001
Any crisis	9434 (20.2)	291 (28.1)	84 (19.6)	162 (36.1)	45 (28.1)	29	2	<.001
Eviction or loss of home	1065 (2.3)	61 (5.9)	10 (2.3)	41 (9.1)	10 (6.2)	18	2	<.001
Death of friend or relative	3610 (7.7)	110 (10.6)	43 (10.0)	57 (12.7)	10 (6.2)	5	2	.07
Financial problem	4341(9.3)	53 (5.1)	15 (3.5)	14 (3.1)	24 (15.0)	38	2	<.001
Family relationship	1911 (4.1)	85 (8.2)	8 (1.9)	38 (8.5)	39 (24.4)	78	2	<.001
**Death Characteristics, No. (%)**								
Cause of death								
Suicide	41 596 (89.0)	980 (94.5)	384 (89.7)	439 (97.8)	S	NA	NA	NA
Other or undetermined	4847 (10.4)	57 (5.5)	44 (10.3)	S	S			
Accidental firearm	279 (0.6)	0	0	0	0			
Means of injury								
Gun or rifle	26 769 (57.3)	529 (51.0)	104 (24.3)	319 (71.0)	106 (66.2)	NA	NA	NA
Sharp or blunt instrument	1202 (2.6)	40 (3.9)	S	S	S			
Poisoning	9551 (20.4)	186 (17.9)	97 (22.7)	62 (13.8)	27 (16.9)			
Fall	778 (1.7)	47 (4.5)	S	S	S			
Other	1549 (3.3)	35 (3.4)	S	S	S			
Unknown	902 (1.9)	11 (1.1)	S	S	S			
Missing	5971 (12.8)	189 (18.2)	130 (30.4)	42 (9.4)	17 (10.6)			
SRF injury location	149 (0.3)	114 (11.0)	106 (24.8)	S	S	NA	NA	NA
LTC/nursing home death location	475 (1.0)	94 (9.1)	88 (20.6)	S	S	NA	NA	NA

Abbreviations: GED, General Education Development; IQR, interquartile range; LTC, long-term care; NA, not applicable; S, suppressed (if <5 to protect confidentiality); SRF, supervised residential facility.

^a^ Test statistic and *P* value for Kruskal-Wallis test or Pearson χ^2^ test comparing across all deaths associated with LTC (living in LTC vs transitioning into or out of LTC vs otherwise associated with LTC).

^b^ The test of significance does not include the missing category in the calculations of the test statistic and *P* value.

**Figure 2.**
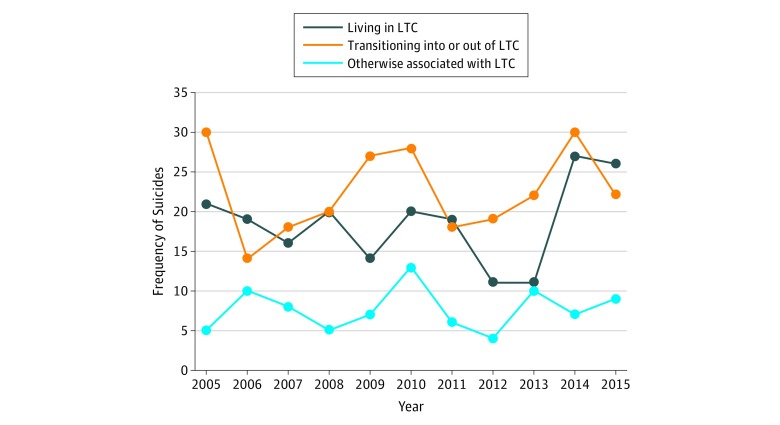
Annual Frequencies of Suicides Associated With Long-term Care (LTC) Among Adults 55 Years and Older, 2005 to 2015 Frequencies are stratified by 3 groups identified by the natural language processing algorithm. Frequencies are limited to the 13 states that began reporting valid data to the National Violent Death Reporting System in 2003 or 2004 (eTable 1 in the Supplement).

Figure 2 shows the number of suicides associated with LTC (living in LTC, transitioning into or out of LTC, or otherwise associated with LTC) from 2005 to 2015. While there is substantial variation, which is expected given the relative rarity of this event, there was no significant change in the number of suicides associated with LTC over time (β = −0.002; *P* = .85). We estimated the agreement between the injury location code SRF (n = 263) and the death location code LTC/nursing home (n = 567) compared with cases that the algorithm identified as occurring in an LTC facility (n = 428) (Table 2 and Table 3). Of the 263 injuries coded as SRF, 106 (40.3%) were identified as occurring in LTC by the algorithm. The agreement between the algorithm and both the injury location code SRF (κ statistic, 0.30; 95% CI, 0.26-0.35) and the death location code LTC/nursing home (κ statistic, 0.17; 95% CI, 0.14-0.20) was poor. Temporarily assuming the NLP algorithm as the criterion standard, the injury location code SRF had a sensitivity of 25% and a specificity of 98% in identifying suicide injuries that occurred in LTC facilities. Finally, eTable 5 in the Supplement lists the distribution of NVDRS injury location codes other than SRF among deaths that occurred in LTC according to the NLP algorithm; almost two-thirds of these cases were coded as occurring at a residential house, apartment, or rooming house.

**Table 2.** Comparison of National Violent Death Reporting System Injury Location SRF With Cases Identified by the NLP Algorithm as Occurring in LTC

SRF	NLP-Identified Suicides, No. (%)		
	Living in LTC	Transitioning Into or Out of LTC or Otherwise Associated With LTC	Non-LTC Deaths
No	428	609	46 722
Yes (n = 263)	106 (24.8)	8 (1.3)	149 (0.3)
No (n = 47 496)	322 (75.2)	601 (98.7)	46 573 (99.7)

Abbreviations: LTC, long-term care; NLP, natural language processing; SRF, supervised residential facility.

**Table 3.** Comparison of NVDRS Death Location Codes for NLP Algorithm–Identified Suicides in an LTC Facility^a^

NVDRS Death Location Code	NLP-Identified Death in LTC, No. (%)	
	SRF Injury Location Code	Non-SRF Injury Location Code
No. of cases	106	322
Inpatient health care facility	18 (17.0)	39 (12.1)
Outpatient facility/emergency department	17 (16.0)	26 (8.1)
LTC/nursing home	30 (28.3)	58 (18.0)
Decedent’s home	22 (20.8)	131 (40.7)
Other or undetermined	19 (17.9)	68 (21.1)

Abbreviations: LTC, long-term care; NLP, natural language processing; NVDRS, National Violent Death Reporting System; SRF, supervised residential facility.

^a^ Death location refers to the NVDRS code of where the person was at the time of his or her death. This may or may not be the same location where the person was when the self-harm occurred (ie, a person could have self-harmed at home or been transported to an emergency department and died there).

## Discussion

This study provides the most comprehensive assessment to date, to our knowledge, of the burden of suicide associated with LTC in the United States. From 2003 to 2015, approximately 2.2% of suicides among adults 55 years and older were associated with LTC in some manner, of which the largest group was persons transitioning into or out of LTC. Using an iterative process, we developed an NLP algorithm that identified more than 400 suicides injuries occurring in an LTC facility. Our findings show that the existing NVDRS location codes substantially underestimate the number of suicides that occur in LTC.

These findings suggest that more can be done to support the mental health of older adults living in these settings. A key element of suicide prevention is the identification of points of engagement before suicide crisis.^4,28^ Almost all US adults want to remain living in their own home as they age, but many report difficulty doing so.^7^ The present results suggest that residential transitions may be an important point of engagement for preventing suicide among older adults. These transitions can be disruptive events both on their own and as a correlate of precipitating changes (eg, becoming widowed or developing difficulties with activities of daily living) that are associated with suicidal behavior.^11,14,29^ Living in LTC or transitioning to LTC is also correlated with a host of characteristics (eg, depression, pain, and frailty) that are established risk factors for suicide.^30,31,32^ As such, living in LTC or LTC transitions may be a marker of underlying risk (ie, functional decline, accumulating medical burden, changes in the quality and quantity of social interaction, or weakening links to personally meaningful social roles) rather than a unique risk factor per se.

While our results are consistent with other findings suggesting that major life transitions are associated with suicide,^3^ they also suggest that the context in which that transition occurs is an important part of why these events can precipitate self-harm.^29^ When considering whether LTC is a potential point of engagement for preventing suicide,^4,5^ we believe that this question benefits from considering LTC settings as not just a physical location but rather as a life transition that happens to involve residence. Residential LTC is also a social setting where people share meals and conversations and can forge meaningful connections,^35^ in addition to accessing health care. Moreover, the experience of living in LTC or transitioning into or out of LTC needs to be considered in a broader social context. These types of transitions involve not just the resident but also his or her family and friends. Indeed, the decision making as to whether, when, and where one will move into residential LTC is almost always undertaken through input of the person and his or her family and clinicians.^36^

### Limitations and Strengths

These study findings should be interpreted in consideration of some limitations. While comprehensive of all suicides in the NVDRS through 2015, our analytic sample is not representative of the entire United States. Several states with large numbers of older adults (eg, Florida) are not represented, although they are scheduled to enter this data system soon.^37^ Most current NVDRS states have centralized coroner/medical examiner systems, which facilitate collection of original documents for abstraction. As additional states enter this system, it is important for researchers to be mindful of variation across states that may introduce bias in reporting (eg, this analysis excluded data from law enforcement narratives specifically because of large amounts of missingness in those fields). The NVDRS only comprises completed suicides and not suicide attempts. Suicide is underreported, and this may be especially true among older adults.^21,22^ We attempted to account for this by including undetermined deaths, but our findings likely remain an underestimate. In addition, it is possible that the NVDRS narratives, while providing more context than is available in almost all other mortality data, are missing potentially relevant information due to the process of data extraction.^38,39^ Because there is substantial state variation as to what constitutes non–nursing home LTC (eg, assisted living) and because there is no systematic annual census of residents living in LTC other than nursing homes,^10^ we could not estimate the cumulative incidence of suicides in these settings. Future studies should leverage data linkages to quantify attempted and completed suicides that occur across all types of LTC settings. Also, while nursing homes are generally regulated at the federal level, there is substantial state variation in the regulation of other forms of residential LTC (eg, independent and assisted living facilities) regarding the requirements for licensing, staffing, facility characteristics, and cost.^40,41,42^ Future research should explore whether this variation relates to geographic variation in suicide risk associated with LTC.

This study also has several strengths. The NLP algorithm was able to efficiently identify cases associated with LTC; this algorithm outperformed the existing NVDRS location codes and provides the most reliable estimate regarding the number of suicides that occur in LTC settings to date, to our knowledge. The success of this algorithm is a function of the amount of information provided in the narratives; future efforts to apply NLP to these data will benefit from continued detailed abstraction of these texts.

## Conclusions

Leaders in the field have continued to call for a shift away from a medicalized paradigm of residential LTC toward institutional practices that instead focus on fostering meaningful interactions between residents, promote engagement in care, and enhance quality of life.^5,11,43^ In addition, existing, scalable programs that support older adults living in the community (eg, home health care, meal assistance, transportation, and community health workers) offer the potential to promote quality of life for older adults who may be considering transitioning into or out of residential LTC. Randomized clinical trials have demonstrated that these programs can positively influence mental health,^44,45,46,47^ and other such trials are underway.^48,49^ There are also ongoing efforts to address barriers to navigating LTC transitions for both residents and family members.^50,51^ These findings emphasize the importance of such efforts for the mental health of older adults.

## Notes

Supplement. **eAppendix.** Additional Description of the National Language Processing (NLP) Algorithm Methods **eTable 1.** Characteristics of States That Prepared and Submitted Data to NVDRS, 2003-2005 **eTable 2.** Characteristics of the Analytic Sample Compared to Deaths Excluded due to Missing Coroner/Medical Examiner (CME) Narrative **eTable 3.** Step-wise Results From Natural Language Processing of Coroner/Medical Examiner NVDRS Narratives **eTable 4.** Heuristic Examples of Case Narratives Describing Suicide Deaths Associated With Long-term Care Annotated After the Final NLP Algorithm **eTable 5.** NVDRS Injury Location Codes Other Than Supervised Residential Facility for Algorithm-Identified Suicides in Long-term Care **eFigure 1.** Detailed Step-wise Development and Training of the NLP Algorithm Using Coroner/Medical Examiner Narratives Available in the National Violent Death Reporting System, 2003-2015 **eFigure 2.** Probability Distribution of Whether Cases in the Testing Data Set Are Associated With LTC as Identified by the NLP Algorithm After the Initial and Final NLP Model: NVDRS, 2003-2015
