# Supplementary material for: Suicide Among Older Adults Living in or Transitioning to Residential Long-term Care, 2003 to 2015
Source: JAMA Netw Open. 2019 Jun 14;2(6):e195627. doi: 10.1001/jamanetworkopen.2019.5627 (PMC6575144; doi:10.1001/jamanetworkopen.2019.5627)
Supplement: Supplement. — eAppendix. Additional Description of the National Language Processing (NLP) Algorithm Methods eTable 1. Characteristics of States That Prepared and Submitted Data to NVDRS, 2003-2005 eTable 2. Characteristics of the Analytic Sample Compared to Deaths Excluded due to Missing Coroner/Medical Examiner (CME) Narrative eTable 3. Step-wise Results From Natural Language Processing of Coroner/Medical Examiner NVDRS Narratives eTable 4. Heuristic Examples of Case Narratives Describing Suicide Deaths Associated With Long-term Care Annotated After the Final NLP Algorithm eTable 5. NVDRS Injury Location Codes Other Than Supervised Residential Facility for Algorithm-Identified Suicides in Long-term Care eFigure 1. Detailed Step-wise Development and Training of the NLP Algorithm Using Coroner/Medical Examiner Narratives Available in the National Violent Death Reporting System, 2003-2015 eFigure 2. Probability Distribution of Whether Cases in the Testing Data Set Are Associated With LTC as Identified by the NLP Algorithm After the Initial and Final NLP Model: NVDRS, 2003-2015 [file jamanetwopen-2-e195627-s001.pdf]

Supplementary Online Content

Mezuk B, Ko TM, Kalesnikava VA, Jurgens D. Suicide among older adults living in or transitioning to residential long-term care, 2003 to 2015. *JAMA Netw Open*. 2019:2(6):e195627. doi:10.1001/jamanetworkopen.2019.5627

eAppendix. Additional Description of the National Language Processing (NLP) Algorithm Methods

eTable 1. Characteristics of States That Prepared and Submitted Data to NVDRS, 2003-2005

eTable 2. Characteristics of the Analytic Sample Compared to Deaths Excluded due to Missing Coroner/Medical Examiner (CME) Narrative

eTable 3. Step-wise Results From Natural Language Processing of Coroner/Medical Examiner NVDRS Narratives

eTable 4. Heuristic Examples of Case Narratives Describing Suicide Deaths Associated With Long-term Care Annotated After the Final NLP Algorithm

eTable 5. NVDRS Injury Location Codes Other Than Supervised Residential Facility for Algorithm-Identified Suicides in Long-term Care

eFigure 1. Detailed Step-wise Development and Training of the NLP Algorithm Using Coroner/Medical Examiner Narratives Available in the National Violent Death Reporting System, 2003-2015

eFigure 2. Probability Distribution of Whether Cases in the Testing Data Set Are Associated With LTC as Identified by the NLP Algorithm After the Initial and Final NLP Model: NVDRS, 2003-2015

This supplementary material has been provided by the authors to give readers additional information about their work.

© 2019 Mezuk B et al. *JAMA Network Open*.

eAppendix. Additional description of the National Language Processing (NLP) algorithm methods

The text below provides additional description of the methods used to generate the NLP algorithm illustrated in **Figure 1** and e**Figure 1**.

(1) Create the initial training data using keyword search. Supervised machine learning requires a labeled ground truth dataset in order to train the classifier algorithm (i.e., it requires known "true positives" and known "true negatives" from which to learn). However, simply annotating a random sample of the approximately 50,000 NVDRS narratives was unlikely to produce a useful training dataset due to the expected rarity of suicides associated with long-term care (LTC). Therefore, we first produced a more limited dataset by searching the coroner/medical examiner (CME) narrative texts using a set of keywords that we identified using an iterative process of identification, manual verification, and correction to filter the narratives.

Inclusion terms (Boolean operator "OR"): We identified a set of 44 keywords: assisted, continuing, convalescent, group home, hospice, institution, institutional, long-term, long term, nursing, old folks, retirement, senior, continu, memory, veterans, VA, housing, homecare, move, moving, respite, rehabilitation, rehab, elder, eldercare, facility, care-giv, caregiv, nurse, adult, daycare, center, supervise, \baid\b, resident, independen, residential, \baged\b, \baging\b, admitt, community, discharge, into a home

*\b b\ denotes word boundaries, which are defined and implemented using the R regular expression library.

Exclusion terms (Boolean operator "NOT"): We also identified a set of seven exclusion terms: hotel, motel, homeless, halfway, residence, jail, prison

These keywords identified 7,806 cases from the CME narratives. Two raters (TK and VK) annotated a random sample of these narratives to construct the initial dataset, with a goal of identifying approximately 100 known "true positives," which is an adequate number of cases for training the algorithm. The initial training dataset consisted of 103 $cases_{LTC}$ (known "true positives") and $264_{non\text{-}LTC}$ (known "true negatives") annotated cases. The strong skew of the data towards non-LTC examples, despite being drawn from a sample of narratives with LTC-related keywords, underscores the difficulty of the classification task.

(2) Train and update the NLP algorithm to identify cases associated with LTC

a) **Train the NLP algorithm:** The initial training dataset of 366 narratives was used to develop a supervised machine learning classifier to identify additional "true positive" cases associated with LTC. The annotated CME text narratives were first converted into a matrix where rows correspond to narratives and columns to the features present in each narrative. Narrative features were then extracted using standard NLP procedures, detailed here:

1. Each narrative was processed into separate sentences and then, within sentences, into separate tokens. This tokenization process separated leading and trailing punctuation from words, such that the phrase "*word1, word2*" becomes three tokens (1) "*word1*", (2) ",", and (3) "*word2*", while intra-token punctuation is kept as-is, e.g., tokens that are standard English contractions such as "don't". Finally, all words were converted to lower-case. This process ensures that the same words can be identified across narrative, as these are written by different NVDRS abstractors.
2. Once the tokenization preprocessing was complete, each narrative was converted into features known as unigrams and bigrams, which are one-word and two-word sequences found within a sentence. For example, the sentence "the patient felt ill" has four unigrams, "the", "patient", "felt", "ill", and three bigrams "the patient", "patient felt", and "felt ill." Bigrams capture local syntactic and narrative

© 2019 Mezuk B et al. *JAMA Network Open*.

structure (e.g., a subject and verb, or an adjective and its modified noun) and are known to highly effective as linguistic features for text classification.

3. Each feature was then assigned to a separate column in the matrix to record the count of how many times a narrative (i.e., a row) contains that unigram or bigram. This data structure is known as a term-document matrix in its general form. As some common words do not aid in distinguishing narrative types (e.g., “the”, “a”, “an”, “to”), we followed common practice in NLP and removed these words as features (columns) from the matrix. We used the full list of words-to-remove from the Python Natural Language Toolkit, commonly known as the NLTK library. We additionally removed all words that only appeared in one narrative, as these are likely to be idiosyncratic language and would not generalize to classifying the other narratives.
4. Not all words are equally useful for classifying narratives; common words are largely uninformative (e.g., most narratives identify the “caliber” of weapon if the decedent died by firearm), while more infrequent words such as “depressed” may be highly informative. Therefore, we applied a common re-weighting technique known as Term Frequency Inverse Document Frequency (TF-IDF) that converts the values according to the formula $f_{d,w} * N/D_w$ where $f_{d,w}$ is the number of times a unigram or bigram (indicated b *w)* appears in document (indicated by *d)*, *N* refers to the total number of documents (narratives) in the collection, and D_w refers to the number of documents in which *w* occurs. This weighting helps improve the efficiency of the classifier.
5. Once all labeled narratives were converted to their numerical vector representations, a Random Forest (RF) classifier was trained to predict the label from the vector. A RF classifier consists of multiple decision trees that are each trained from a random subset of the data to predict the external data (that is, the data not included in the particular random subset). By using multiple trees (1,500 in our case), the RF classifier is robust to overfitting even in small datasets such as ours. We used the default parameter choices for the RF implementation in Scikit-learn, except that we use 1,500 trees.
6. Ultimately, the performance of the RF classifier on the testing dataset (i.e., those narratives that were not labeled as “true positive” or “true negative” by the authors) is estimated through a process known as cross-validation. The labeled dataset is partitioned into *k* disjoint subsets, where *k*-1 are used to train the RF model and the kth partition is used to test the model’s predictions with the actual labels; this process repeats until all of the partitions have been used as test data and the average score of all test partitions. Here, we use *k*=5 and evaluate using F1, which is the harmonic mean of *precision* (mathematically-equivalent to *positive predictive value*) and *recall* (mathematically-equivalent to *sensitivity*). F1 has a possible range 0 to 1.0, with higher values indicating higher accuracy. The classifier attained an F1 of 0.880 using the initial annotated training dataset, which indicates high performance at distinguishing narratives associated with LTC and those that are not.
7. Machine learning classifiers can be used to estimate the probability of an instance belonging to a class, e.g., the probability that a narrative is associated with LTC. Inspecting these probabilities allows us to identify cases where the classifier is least-certain about the decision, i.e., those instances whose probabilities that are close to 0.50. **eFigure 2a** shows the probability distribution of the classifier at this step. Using the default threshold probability of 0.50 to determine true positive/true negative assignment, the algorithm identified 943 narratives potentially associated with LTC at this step.

b) **Manually annotate a sample of the output of the algorithm and update the NLP training data**: Next, narratives were sampled from across the full probability distribution of the classifier’s predictions from Step 2

© 2019 Mezuk B et al. *JAMA Network Open*.

(**eFigure 2a**). Note that this range is heavily right-skewed, indicating the model was not very certain of "true positive" case status at this stage, likely due to limited size of the dataset and the presence of ambiguous linguistic features in the narratives.

Five samples (totaling 41 cases) were selected from across the probability range for manual review to assign known "true positive" and known "true negative" status (e.g., 10 cases from the probability range [0.2, 0.3)). Multiple raters (TK, VK, BM, and DJ) independently reviewed a random sample of these cases and annotated/classified them as "true positive" or "true negative." The initial amount of interrater agreement as to whether a case was a "true positive" or "true negative" was high (Kappa=0.7). All discordant cases were discussed, and status was decided by consensus agreement, resulting in 14 known "true positive" and 27 known "true negative" cases. These annotated cases were then added to the training dataset.

By providing this type of iterative feedback the algorithm learned the features (i.e., types of words/phrases, the context and structure of those words/phrases) of known "true positive" cases, which improved the sensitivity and specificity of the classifier. Further, by selecting items from near the confidence range, especially near the decision boundary of 0.50, the newly-annotated cases potentially allow the model to resolve ambiguity about whether certain linguistic features are associated with one class or another.

(3) Finalize the algorithm and characterize cases

a) **Final iteration of the NLP model:** Step 2 was repeated with the updated training set (n=117 known "true positives" and 290 known "true negative"). **eFigure 2b** shows the probability distribution at this step, which is bimodal compared to **eFigure 2a** and illustrates the improved discriminating power of the algorithm. The classifier performance remained consistent with that of Step 2. The final NLP algorithm had a Precision of 0.91 and Recall of 0.86, which yielded an F1 of 0.88 using an identical cross-validation setup. Together, these results point to an increased ability to determine the previously ambiguous instances were associated to LTC without a decrease in performance.

Using the standard classification threshold probability of 0.50, the algorithm identified 1200 suicides $cases_{LTC}$. Compared to the algorithm at Step 2, this final algorithm had a sensitivity of 67.7% (it identified 388 new "true positive" cases) and a specificity of 99.7% (it reclassified 119 initial cases as "true negatives"). All cases with a probability of ≤0.50 at this step were assigned the status of "not being associated with LTC"

b) **Manual review and characterization of the final case set:** Two raters (VK and TK) each manually reviewed 50% (n=600) $cases_{LTC}$ identified by the algorithm and classified them as one of the following: (a) decedent was residing in residential LTC at the time of their death, (b) decedent was anticipating and/or in the process of transitioning into (or out of) LTC at the time of their death; (c) decedent was caring for a family member/friend who was living in/transitioning to LTC at the time of their death; or reclassified as (d) not associated with LTC (i.e., false positives). These raters used the process developed at Step 3 for classifying cases association with LTC.

Once the 1,200 narratives were annotated, this four-level categorical indicator variable was merged back with the existing NVDRS quantitative data to characterize the descendants, as illustrated in **Tables 1** and **2**.

Heuristic examples of CME narratives indicating the four types of cases identified by the NLP algorithm

eTable 4 provides heuristic examples of narratives that were written by the authors (that is, are not actual narratives from the restricted-access NVDRS data) but are instead descriptions based on multiple actual narratives from each of the four groupings identified by the NLP algorithm: (a) decedent was residing in residential LTC at the time of their death, (b) decedent was in the process of transitioning into (or out of) LTC

© 2019 Mezuk B et al. *JAMA Network Open*.

at the time of their death; (c) decedent was otherwise associated with LTC at the time of their death; or (d) not associated with LTC.

Actual narratives are not provided to preserve decedent confidentiality and in accordance with the CDC data use agreement. These examples are provided simply to give the reader a better understanding of the types of information provided in the CME narratives which the algorithm uses to identify cases.

© 2019 Mezuk B et al. *JAMA Network Open*.

eTable 1. Characteristics of states that prepared and submitted data to NVDRS 2003 – 2005

State	Years in NVDRS	Suicides aged ≥55 Total N	Undetermined deaths aged ≥55 Total N	Suicides $cases_{LTC}$† N (row %)	Type of Coroner or Medical Examiner (ME) System	Deaths with Non-Missing Narratives	Mean Narrative Length in Characters (SD)
Alaska	2003-2015	436	77	7 (1.6)	Centralized ME	436	809.17 (420.82)
Arizona	2015	477	35	5 (1.0)	County-based ME	453	1,155.19 (583.24)
Colorado*	2004-2015	3,319	233	94 (2.8)	County-based Coroner	3,076	624.34 (421.5)
Connecticut	2015	153	12	S	County-based ME & Coroner	156	476.31 (223.86)
Georgia	2004-2015	4,552	335	49 (4.7)	County-based ME & Coroner	3,520	442.55 (356.94)
Hawaii*	2015	87	15	S	County-based ME & Coroner	81	714.77 (454.7)
Kansas	2015	159	S	S	County-based Coroner	156	482.67 (303.95)
Kentucky	2005-2015	2,467	204	28 (1.1)	County-based Coroner	2,334	223.12 (192.54)
Maine	2015	98	S	S	Centralized ME	100	344.68 (113.21)
Maryland	2003-2015	3,399	1,255	74 (2.1)	Centralized ME	3,464	517.22 (261.9)
Massachusetts	2003-2015	2,385	418	44 (1.8)	Centralized ME	2,395	392.73 (178.49)
Michigan	2014-2015	1,069	150	10 (0.9)	County-based ME	952	418.58 (216.82)
Minnesota	2015	229	32	8 (3.4)	County-based ME & Coroner	206	689.13 (458.02)
New Hampshire	2015	78	S	S	Centralized ME	79	908.29 (301.51)
New Jersey	2003-2015	3,188	300	56 (1.7)	County-based ME	3,127	601.75 (462.7)
New Mexico	2005-2015	1,549	177	53 (3.3)	Centralized ME	1,581	701.29 (434.36)
New York	2015	618	51	S	County-based ME and Coroner	596	435.82 (360.77)
North Carolina	2004-2015	4,821	171	69 (1.4)	Centralized ME	4,878	570.8 (162.75)
Ohio	2011-2015	2,848	238	61 (2.1)	County-based ME and Coroner	2,738	508.2 (204.04)
Oklahoma	2004-2015	2,390	261	71 (2.9)	Centralized ME	2,454	489.43 (215.4)
Oregon*	2003-2015	3,253	251	112 (3.3)	Centralized ME	3,284	595.83 (390.46)
Rhode Island	2004-2015	477	93	16 (3.2)	Centralized ME	493	501.63 (336.91)
South Carolina	2003-2015	2,701	92	33 (1.2)	County-based Coroner	2,476	297.74 (215.86)
Utah	2005-2015	1,672	448	28 (1.6)	Centralized ME	1,683	740.19 (305.25)
Vermont*	2015	47	6	S	Centralized ME	48	593.92 (266.66)
Virginia	2003-2015	4,206	208	111 (2.6)	Centralized ME	4,317	390.18 (255.95)
Wisconsin	2004-2015	2,622	157	96 (3.5)	County-based ME & Coroner	2,676	578.6 (401.92)

Deaths were assigned to the state where the death occurred and which abstracted the data, i.e. a given state may have abstracted data for the death of an individual who lived in another state.

†Refers to deaths associated with LTC as determined by the NLP algorithm. S: Cells <5 are suppressed to protect confidentiality per CDC Data Use Agreement.

© 2019 Mezuk B et al. *JAMA Network Open.*

*State with Physician-Assisted Suicide/Death with Dignity Laws: Vermont (passed in May 2013, practiced since 2017); Colorado (December, 2016); Oregon (October, 1997); Hawaii (January, 2019).

© 2019 Mezuk B et al. *JAMA Network Open*.

eTable 2. Characteristics of the analytic sample compared to deaths excluded due to missing Coroner/Medical Examiner (CME) narrative

	Analytic sample N (col %)	Excluded because of missing CME narrative N (col %)	Kruskal-Wallis or Pearson's $\mathcal{X}^2$ test ($\mathcal{X}^2$, DF) p-value
Total N	47,759	2,578	
Injury location code: Supervised Residential Facility (SRF)	263 (0.6)	S	(6, 1) 0.01
Place of death code			(1713, 7) 0.00
Inpatient healthcare facility	3,542 (7)	330 (13)	
Outpatient facility/ED	3,016 (6)	156 (6)	
Dead on arrival	604 (1)	66 (3)	
Hospice facility	172 (0.4)	16 (0.6)	
Nursing home/Long-term care	569 (1.2)	64 (3)	
Decedent's home	30,852 (65)	1,321 (51)	
Other/Undetermined	8,693 (18)	402 (16)	
Unknown/Missing	311(0.7)	223 (9)	
Age (median, IQR)	64 (15)	66 (17)	(194, 48) 0.00
% Male	36,995 (78)	1,989 (77)	(0, 1) 0.85
% Non-Hispanic White	42,961 (90)	2,285 (89)	(5, 1) 0.03
Education			(94, 3) 0.00
< High School	5,325 (11)	344 (13)	
HS or GED	11,879 (25)	531 (21)	
> High School	12,378 (26)	526 (20)	
Unknown/Missing	18,177 (38)	1177 (46)	
Marital Status			(245, 4) 0.00
Married/In relationship	21,020 (44)	1,159 (45)	
Single/Never married	5,165 (11)	207 (8)	
Widowed	7,503 (16)	408 (16)	
Divorced/Separated	13,261 (28)	653 (25)	
Unknown/Missing	810 (2)	151 (6)	
Depressed Mood	17,257 (36)	261 (10)	(728, 1) 0.00
Physical Health Problems	18,264 (38)	336 (13)	(666, 1) 0.00
History of Suicide Ideation	5,749 (12)	65 (3)	(216, 1) 0.00
Means of Injury			
Gun or rifle	27,298 (57)	1,478 (57)	(274, 8) 0.00
Sharp or blunt instrument	1,242 (3)	39 (2)	
Poisoning	9,737 (20)	315 (12)	
Fall	825 (2)	27 (1)	

© 2019 Mezuk B et al. *JAMA Network Open*.

Drowning	718 (2)	28 (1)	
Fire or Burns	287 (0.6)	13 (0.5)	
Motor or Transport Vehicle	354 (0.7)	15 (0.6)	
Other	225 (0.5)	16 (0.6)	
Unknown or Missing	7,073 (15)	647 (25)	
Recent Crisis	5,477 (12)	100 (4)	(142, 1) 0.00
Any Crisis	9,725 (20)	136 (5)	(352, 1) 0.00
Eviction or loss of home	1,126 (3)	13 (0.5)	(37, 1) 0.00
Death of friend or relative	3,720 (8)	43 (2)	(132, 1) 0.00
Financial Problem	4,394 (9)	62 (3)	(139, 1) 0.00
Family Relationship Issue	1,996 (4)	46 (2)	(35, 1) 0.00

S: Cells <5 are suppressed to protect confidentiality per CDC Data Use Agreement.

© 2019 Mezuk B et al. *JAMA Network Open*.

eTable 3. Step-wise Results from Natural Language Processing of Coroner/Medical Examiner NVDRS Narratives

**Steps in developing NLP algorithm to identify LTC cases**	**Cases associated with Long-term Care (LTC)**			**Total n of LTC cases* at each step**
	Suicide in LTC	*Transitioning to LTC*	*Otherwise related to LTC*	
1. Keywords-based search	90	11	2	**103**
2. Train NLP Algorithm	7	6	1	**14**
3. Finalize NLP Model	331	432	157	**920**
**Total N associated with LTC**	**428**	**449**	**160**	**1037**

*Non LTC cases were also included in each step of NLP development and training (Step 1: 264 $cases_{non\text{-}LTC}$; Step 2: 27 $cases_{non\text{-}LTC}$; Step 3: 280 $cases_{non\text{-}LTC}$)
NLP=Natural Language Processing algorithm. NVDRS=National Violent Death Reporting System

© 2019 Mezuk B et al. *JAMA Network Open*.

eTable 4. Heuristic examples of case narratives describing suicide deaths associated with long-term care annotated after the final NLP algorithm

**Type of Case**<br>**Determined by manual review**	**Heuristic CME Narratives**
Decedent was living in an LTC facility	Victim was found in her assisted living center apartment. She had moved to this location recently due to declining health and increased difficulty getting around.
Decedent was transitioning into LTC	Victim was found at his residence. Victim had been experiencing difficulty walking and carrying out daily activities. Victim had stated that he was believed death would be preferable to living in a nursing home.
Decedent was otherwise associated with LTC	Victim was found at home. The victim's spouse was in a nursing home with dementia, and victim had expressed distress over this.
Death was indexed as not associated with LTC	Victim was found at his home. Victim had a history of chronic medical problems including pain. Victim had recently spoken to a nurse about his health concerns.
Note: These narratives were written by the authors to preserve decedent confidentiality but are based on actual Coroner/Medical Examiner (CME) NVDRS narratives.	

© 2019 Mezuk B et al. *JAMA Network Open*.

eTable 5. NVDRS injury location codes other than Supervised Residential Facility for algorithm-identified suicides in long-term care

**NVDRS injury location codes**	**NLP-identified death in LTC but had an NVDRS injury location code other than SRF** N (column %)
No. of cases	322
House, apartment, rooming house, including driveway, porch, garage, yard, etc.	200 (62)
Public/commercial spaces, bridges, highways, stores, parking lots, places of prayer, etc.	12 (4)
Hospital or medical facility	71 (22)
Farm, park or natural area	20 (6)
Hotel/motel, other or unknown location	19 (6)

Injury location code refers to the location where the self-harm occurred, which may or may not be the same location of where the death occurred (i.e., a person could have self-harmed at home, been transported to an Emergency Department where they then died).

SRF: Supervised Residential Facility. NLP: Natural Language Processing algorithm. NVDRS: National Violent Death Reporting System. LTC: Long-term care.

© 2019 Mezuk B et al. *JAMA Network Open*.

eFigure 1. Detailed step-wise development and training of the NLP algorithm using coroner/medical examiner narratives available in the National Violent Death Reporting System: 2003 – 2015.

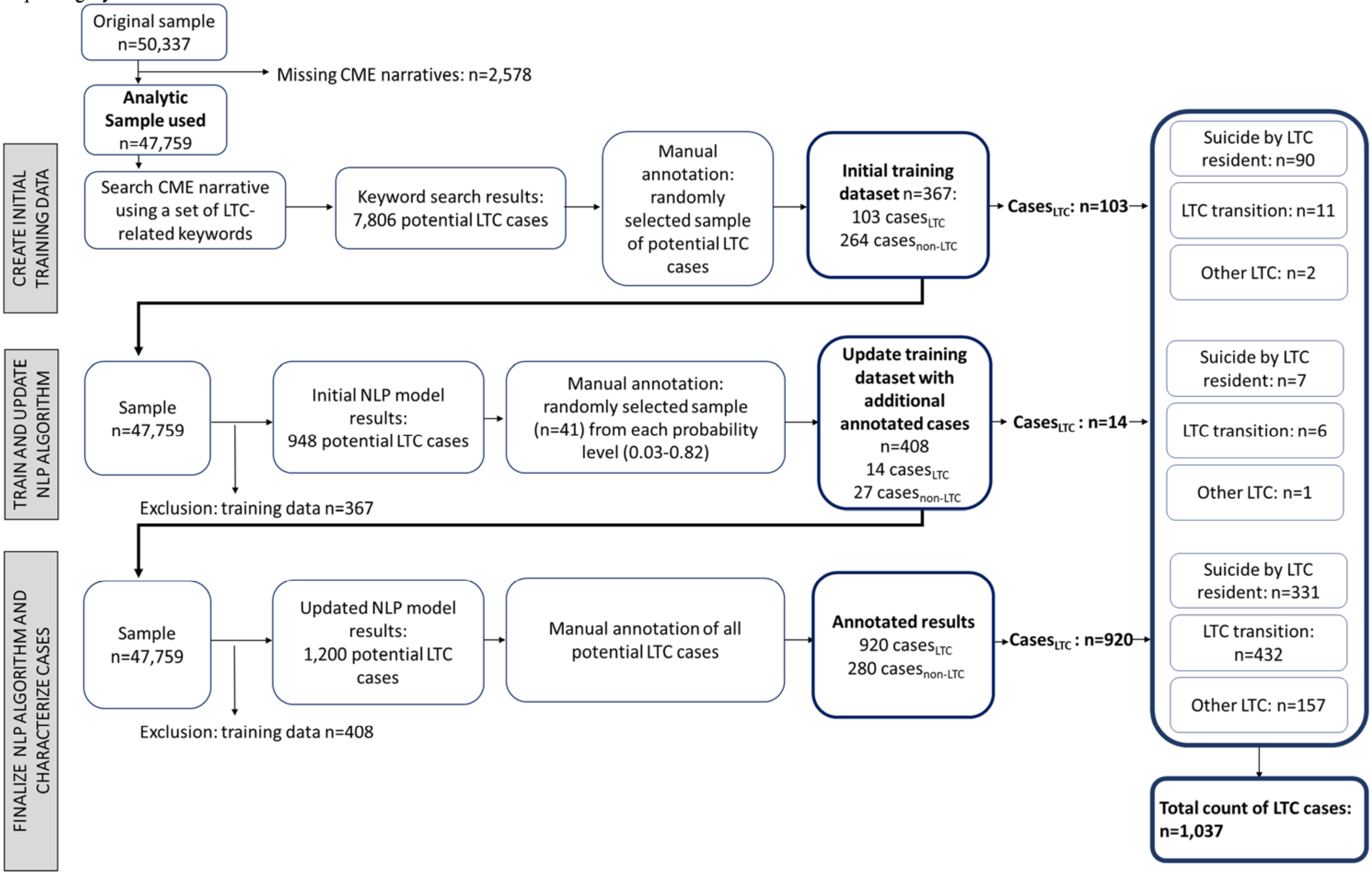

© 2019 Mezuk B et al. *JAMA Network Open*.

Caption: Detailed step-wise development and training of NLP algorithm, using coroner/medical examiner narratives of decedents aged ≥55 years old (n=47,759) in the National Violent Death Reporting System (NVDRS) between 2003 – 2015.

© 2019 Mezuk B et al. *JAMA Network Open*.

eFigure 2a-b. Probability distribution of whether cases in the testing dataset are associated with LTC as identified by the NLP algorithm after the initial and final NLP model: NVDRS 2003 – 2015

Figure 2a

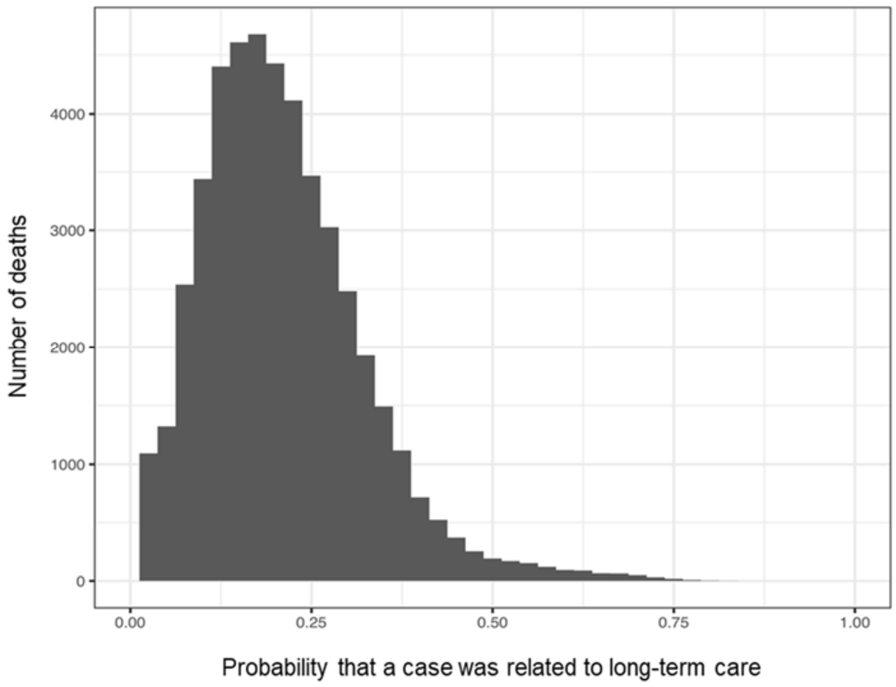

Figure 2b

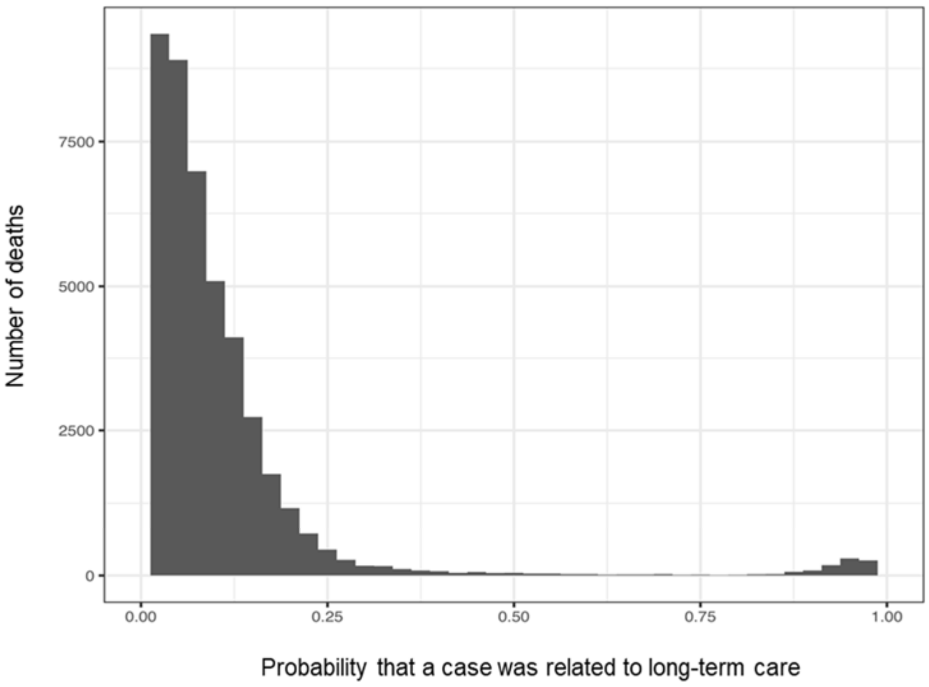

Caption: eFigure 2a illustrates the distribution of probabilities that a case is associated with LTC after the first iteration of the NLP algorithm. It identified 943 potential LTC cases with a probability >0.5. Figure 2b shows the distribution of probabilities that a case is associated with LTC after the final iteration of NLP algorithm. It identified 1200 potential LTC cases with a probability >0. 50. The bimodal distribution of eFigure 2b relative to eFigure 2a shows the improved certainty of case classification with the iterative refinement of the model.

© 2019 Mezuk B et al. *JAMA Network Open*.
